# 2017至2023年邯郸市肺癌死亡趋势及减寿分析

**DOI:** 10.3779/j.issn.1009-3419.2025.106.15

**Published:** 2025-06-30

**Authors:** Nianzhen FANG, Yang ZHAO, Yang YANG

**Affiliations:** 056000 邯郸，邯郸市疾病预防控制中心; Handan Prefectural Center for Disease Control and Prevention, Handan 056000, China

**Keywords:** 肺肿瘤, 去死因期望寿命, Fulfillment指数, 潜在减寿年数, Lung neoplasms, Cause eliminated life expectancy, Fulfillment index, Potential years of life lost

## Abstract

**背景与目的** 肺癌位居我国癌症死亡的首位，严重损害居民的生命健康。通过分析邯郸市2017至2023年肺癌死亡趋势及其导致的寿命损失情况，为防治策略的制定提供数据支撑。**方法** 收集2017至2023年邯郸市因肺癌导致的死亡资料，应用Excel 2010、SPSS 26.0和Joinpoint 4.9.0.0软件分析肺癌死亡率、平均年度变化百分比（average annual percentage change, AAPC）、去死因期望寿命（cause eliminated life expectancy, CELE）、去死因期望寿命增长年（potential gains in life expectancy, PGLEs）、Fulfillment指数、潜在减寿年数（potential years of life lost, PYLL）、潜在减寿率（potential years of life lost rate, PYLLR）、标化潜在减寿率（standardized potential years of life lost rate, SPYLLR）等指标。**结果** 2017至2023年邯郸市肺癌标化死亡率呈下降趋势（AAPC=-7.10%, *P*<0.01）。肺癌CELE增长了2.49岁（AAPC=0.48%, *P*<0.05）；寿命损失率下降21.43%（AAPC=-4.61%, *P*<0.05）。2017至2023年肺癌的Fulfillment指数随着年龄的增长而增加。2017至2023年邯郸市因肺癌导致的PYLL为134,219.75人年，PYLLR为2.03‰，标化潜在减寿年数（standardized potential years of life lost, SPYLL）为98,735.63人年，SPYLLR为1.49‰，历年PYLLR和SPYLLR均呈下降趋势（AAPC=-6.34%和-9.34%，P均<0.01）。**结论** 2017至2023年邯郸市肺癌标化死亡率呈下降趋势，肺癌对期望寿命的影响有所减轻。不同年龄及分性别的肺癌死亡率存在差异，应针对男性和高年龄组人群做好肺癌的防控工作。

## Abstract (en)

**Background and objective** Lung cancer ranks as the foremost cause of cancer-related deaths in China, significantly undermining population health and longevity. By analyzing the trends of death and years of life lost caused by lung cancer in Handan from 2017 to 2023, data support is provided for the formulation of prevention and treatment strategies.**Methods** Lung cancer death data in Handan during 2017-2023 were collected. Excel 2010, SPSS 26.0 and Joinpoint 4.9.0.0 were used to analyze the mortality rate of lung cancer, average annual percentage change (AAPC), cause eliminated life expectancy (CELE), potential gains in life expectancy (PGLEs), Fulfillment index, potential years of life lost (PYLL), potential years of life lost rate (PYLLR), and standardized potential years of life lost rate (SPYLLR).**Results** The standardized mortality rate of lung cancer in Handan from 2017 to 2023 showed a decreasing trend (AAPC=-7.10%, *P*<0.01). The CELE of lung cancer increased by 2.49 years (AAPC=0.48%, *P*<0.05). The life loss rate decreased by 21.43% (AAPC=-4.61%, *P*<0.05). The Fulfillment index by lung cancer increased with the growth of age from 2017 to 2023. The PYLL, PYLLR, standardized potential years of life lost (SPYLL) and SPYLLR of lung cancer during 2017 to 2023 were 134,219.75 person years, 2.03‰, 98,735.63 person years, and 1.49‰, respectively. The annual PYLLR and SPYLLR showed a decreasing trend (AAPC=-6.34%, -9.34%, respectively, *P*<0.01).**Conclusion** The standardized mortality rate of lung cancer in Handan from 2017 to 2023 showed a decreasing trend, and the impact of lung cancer on life expectancy decreased. The mortality rates of lung cancer showed significant differences among different ages and genders. It is necessary to take good measures to prevent and control lung cancer in males and higher age groups.

肺癌是严重威胁人民群众健康的重要恶性肿瘤，造成了严重的疾病负担^[1]^。依据中国肿瘤登记平台数据^[2]^估计，2022年我国肺癌新发病例106.06万，死亡病例73.33万，其发病和死亡均位居恶性肿瘤的首位。研究^[3]^显示，吸烟是导致肺癌发生的主要危险因素，在肺癌患病风险中，吸烟者要比未吸烟者高10-40倍。有报告^[4]^指出，恶性肿瘤死亡率的增加对期望寿命的增长产生了负向影响。潜在减寿年数（potential years of life lost, PYLL）作为衡量疾病负担的重要指标，可用于评估某种疾病对寿命的影响程度^[5]^。本研究利用邯郸市常住居民死因监测数据，分析2017至2023年邯郸市肺癌死亡特征及减寿情况，为优化和普及肺癌防治措施、提高人口期望寿命提供数据支撑。

## 1 资料与方法

### 1.1 资料来源

本研究数据来源于中国死因监测登记系统 2017年1月1日至2023年12月31日常住居民死亡资料。人口资料来自于当地的统计局。根据国际疾病分类-10（international Classification of diseases-10, ICD-10）^[6]^的标准进行编码，肺癌的编码范围为C33-C34.9。

### 1.2 质量控制

全市各级各类医疗机构通过中国疾病预防控制系统的人口死亡信息登记系统对死亡病例实时报告。由医疗机构、区和市疾病预防控制中心对死亡数据进行三级质量审核。定期与公安局、民政局等部门进行数据比对工作，并开展漏报调查，确保死亡数据的完整性、准确性和可靠性，2017至2023年全市死因报告质量符合国家相关标准，错误编码率<5%。

### 1.3 统计学处理

采用Excel 2010、SPSS 26.0软件进行统计分析，计算2017至2023年邯郸市居民的肺癌粗死亡率、标化死亡率，分性别及分年龄段的期望寿命^[7]^、去死因期望寿命（cause eliminated life expectancy, CELE）^[8]^、去死因期望寿命增长年（potential gains in life expectancy, PGLEs）^[9]^、Fulfillment指数^[10,11]^、PYLL等指标，率的标化选用全国2000年第五次普查人口的标准数据。采用Joinpoint 4.9.0.0软件进行趋势分析，计算平均年度变化百分比（average annual percentage change, AAPC），该模型通过拟合肺癌死亡率的对数线性模型，采用蒙特卡洛置换检验方法判断连接点的个数、位置及相应的P值。*P*<0.05为差异具有统计学意义。

## 2 结果

### 2.1 肺癌死亡率及变化趋势

2017至2023年邯郸市因肺癌死亡人数为16,735人，粗死亡率为25.28/10万，标化死亡率为16.58/10万。男性因肺癌死亡人数为11,719人，女性因肺癌死亡人数为5016人，男性肺癌标化死亡率为25.89/10万，女性标化死亡率为8.93/10万。2017至2023年邯郸市肺癌标化死亡率整体呈现下降趋势（AAPC=-7.10%, *P*<0.01）。不同年份之间进行比较，男性肺癌的粗死亡率及标化死亡率始终高于女性；不同性别之间肺癌标化死亡率变化趋势与总体一致，男女标化死亡率AAPC分别为-6.28%和-8.53%（*P*均<0.05），见表1。

**表1:** 2017至2023年邯郸市不同性别居民肺癌死亡变化情况

Year	Male			Female			Total	
	Crude mortality rate (/10^5^)	ASMRC (/10^5^)		Crude mortality rate (/10^5^)	ASMRC (/10^5^)		Crude mortality rate (/10^5^)	ASMRC (/10^5^)
2017	38.43	33.59		18.97	13.31		28.78	22.54
2018	36.35	29.83		15.24	10.06		25.79	19.04
2019	35.86	27.45		14.99	9.33		25.50	17.48
2020	34.58	24.94		15.09	8.79		24.92	16.09
2021	34.21	24.18		13.87	7.76		24.04	15.20
2022	31.82	21.67		12.45	6.91		22.00	13.50
2023	36.72	23.68		15.43	7.91		25.91	14.93
AAPC (%)	-1.59	-6.28		-3.86	-8.53		-2.44	-7.10
t	-1.56	-5.68		-2.01	-4.58		-1.94	-5.50
P	0.18	<0.01		0.10	0.01		0.11	<0.01

ASMRC: age-standardized mortality rates by Chinese standard population; AAPC: average annual percentage change.

### 2.2 不同年龄别肺癌死亡率及变化趋势

2017至2023年邯郸市总人群的肺癌死亡率在50~岁之前处于较低水平，之后随着年龄的增长而增加，在85~岁年龄组时肺癌的死亡率达到高峰；在45~岁组、50~岁组、55~岁组、60~岁和70~岁年龄组中，各年份间肺癌的死亡率呈下降趋势（*P*<0.05），其他年龄组肺癌的死亡率变化趋势无统计学意义（*P*>0.05），见表2。

**表2:** 2017至2023年邯郸市不同年龄居民肺癌死亡变化情况（/10万）

Year	0~	5~	10~	15~	20~	25~	30~	35~	40~	45~	50~	55~	60~	65~	70~	75~	80~	85~
2017	0.00	0.00	0.00	0.69	0.21	0.41	2.49	2.28	4.61	12.30	31.13	35.08	72.14	168.96	203.13	218.82	317.91	542.35
2018	0.00	0.11	0.00	0.70	0.11	0.42	2.35	2.33	2.79	10.08	23.44	34.86	71.02	143.45	181.61	163.79	256.63	379.33
2019	0.00	0.00	0.00	0.25	0.00	0.33	1.03	2.90	2.85	8.96	18.57	33.29	60.85	77.61	193.39	220.01	267.46	417.81
2020	0.00	0.00	0.00	0.00	0.00	0.88	0.53	1.93	3.88	6.59	17.75	25.86	67.46	87.97	165.43	195.24	263.88	272.10
2021	0.00	0.00	0.00	0.24	0.22	0.94	1.55	2.06	5.97	5.37	16.27	29.25	54.28	91.61	164.70	181.60	193.44	218.53
2022	0.00	0.00	0.11	0.16	0.00	0.44	0.61	1.62	3.94	5.87	14.15	23.76	52.98	72.18	114.46	204.99	219.80	238.30
2023	0.00	0.00	0.00	0.14	0.24	0.25	0.81	1.73	3.23	4.42	13.63	25.87	48.19	94.94	135.39	193.75	290.08	329.25
AAPC (%)	-	-	-	-	-	-1.22	-18.30	-6.55	1.30	-15.34	-12.81	-6.26	-6.60	-9.96	-7.89	-0.39	-3.19	-10.40
t	-	-	-	-	-	-0.12	-2.19	-2.47	0.23	-9.33	-8.17	-3.98	-5.82	-2.29	-3.97	-0.18	-1.04	-2.39
P	-	-	-	-	-	0.91	0.08	0.06	0.83	<0.01	<0.01	0.01	<0.01	0.07	0.01	0.87	0.35	0.06

### 2.3 肺癌CELE

邯郸市居民期望寿命从2017年的77.44岁增长到2023年的80.03岁，增长了2.59岁，其中男性增长了2.38岁，女性增长了2.59岁。肺癌CELE由2017年的77.98岁增长到2023年的80.47岁，增长了2.49岁（AAPC=0.48%, *P*<0.05）；寿命损失率由2017年的0.70%减少到2023年的0.55%，下降了21.43%（AAPC=-4.61%, *P*<0.05）。2017至2023年邯郸市男性居民肺癌CELE均低于女性居民，男性的寿命损失率均高于女性，见表3。

**表3:** 2017至2023年邯郸市期望寿命、肺癌CELE、PGLEs和寿命损失率的变化趋势

Year	Life expectancy (yr)				CELE (yr)				PGLEs (yr)				Life loss rate (%)		
	Male	Female	Total		Male	Female	Total		Male	Female	Total		Male	Female	Total
2017	74.44	80.69	77.44		75.05	81.11	77.98		0.61	0.42	0.54		0.82	0.52	0.70
2018	75.23	81.71	78.38		75.83	82.05	78.88		0.60	0.34	0.50		0.80	0.42	0.64
2019	75.50	81.47	78.41		76.03	81.77	78.84		0.53	0.30	0.43		0.70	0.37	0.55
2020	76.57	82.49	79.48		77.11	82.80	79.93		0.54	0.31	0.45		0.71	0.38	0.57
2021	76.55	83.27	79.86		77.10	83.57	80.31		0.55	0.30	0.45		0.72	0.36	0.56
2022	76.10	82.80	79.39		76.54	83.05	79.76		0.44	0.25	0.37		0.58	0.30	0.47
2023	76.82	83.28	80.03		77.35	83.59	80.47		0.53	0.31	0.44		0.69	0.37	0.55
AAPC (%)	0.47	0.51	0.51		0.44	0.49	0.48		-3.53	-5.30	-4.10		-3.97	-5.96	-4.61
t	4.25	5.33	5.18		3.88	5.14	4.83		-2.34	-2.55	-2.60		-2.71	-2.92	-3.08
P	0.01	<0.01	<0.01		0.01	<0.01	0.01		0.07	0.05	0.05		0.04	0.03	0.03

CELE: cause eliminated life expectancy; PGLEs: potential gains in life expectancy.

### 2.4 肺癌的Fulfillment指数分析

肺癌的Fulfillment指数随着年龄的增长，人群潜在寿命损失程度呈现高年龄段上升的趋势。在80~岁年龄组中，历年Fulfillment指数达到最高值。不同年份之间进行比较，在45~岁组、50~岁组、55~岁年龄组居民中，Fulfillment指数呈下降趋势；其余年龄组趋势变化不明显，见图1。

**图1:**
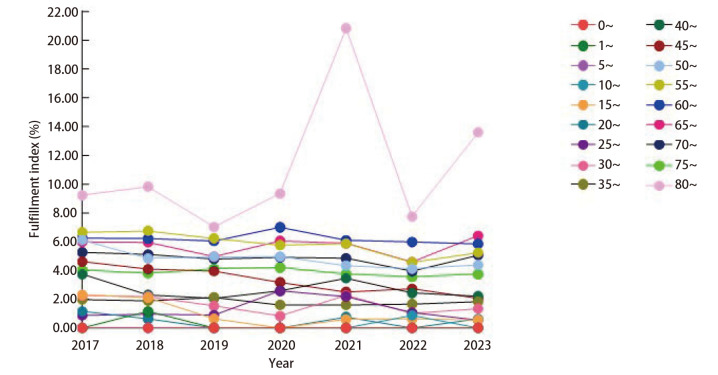
2017至2023年邯郸市居民因肺癌死亡的Fulfillment指数分布及其变化情况

### 2.5 肺癌导致的寿命损失情况

2017至2023年邯郸市居民因肺癌导致的寿命损失总量为134,219.75人年[标化潜在减寿年数（standardized potential years of life lost, SPYLL）为98,735.63人年]，潜在减寿率（potential years of life lost rate, PYLLR）为2.03‰[标化潜在减寿率（standardized potential years of life lost rate, SPYLLR）为1.49‰]，历年PYLLR（AAPC=-6.34%, *P*<0.01）和SPYLLR均呈下降趋势（AAPC=-9.34%, *P*<0.01）；男性PYLL为96,924.25人年（SPYLL为72,508.55人年），PYLLR为2.93‰（SPYLLR为2.19‰），历年PYLLR（AAPC=-5.68%, *P*<0.01）和SPYLLR均呈下降趋势（AAPC=-8.49%, *P*<0.01）；女性PYLL为37,295.50人年（SPYLL为27,409.97人年），PYLLR为1.13‰（SPYLLR为0.83‰），历年PYLLR（AAPC=-7.57%, *P*<0.01）和SPYLLR均呈下降趋势（AAPC=-11.14%, *P*<0.01），且无论是PYLL、PYLLR、SPYLLR，女性均低于男性，见表4。

**表4:** 2017至2023年邯郸市因肺癌导致的寿命损失情况

Year	Male					Female					Total			
	PYLL (person-year)	PYLLR (‰)	SPYLL (person-year)	SPYLLR (‰)		PYLL (person-year)	PYLLR (‰)	SPYLL (person-year)	SPYLLR (‰)		PYLL (person-year)	PYLLR (‰)	SPYLL (person-year)	SPYLLR (‰)
2017	17,028.75	3.56	14,530.58	3.04		7125.75	1.51	5970.43	1.27		24,154.50	2.54	20,316.58	2.14
2018	15,671.00	3.30	13,265.17	2.79		5752.50	1.21	4694.40	0.99		21,423.50	2.25	17,779.98	1.87
2019	14,024.75	2.92	10,089.93	2.10		5429.75	1.15	3967.33	0.84		19,454.50	2.04	13,863.54	1.46
2020	13,627.75	2.83	9580.80	1.99		5468.75	1.16	3799.16	0.80		19,096.50	2.00	13,203.66	1.38
2021	13,788.50	2.93	10,280.88	2.18		5003.50	1.06	3381.01	0.72		18,792.00	2.00	13,471.65	1.43
2022	11,164.00	2.41	8103.43	1.75		4192.25	0.88	3086.55	0.65		15,356.25	1.63	11,064.18	1.17
2023	11,619.50	2.54	8182.70	1.79		4323.00	0.92	2910.34	0.62		15,942.50	1.72	10,989.75	1.18
Total	96,924.25	2.93	72,508.55	2.19		37,295.50	1.13	27,409.97	0.83		134,219.75	2.03	98,735.63	1.49
AAPC (%)		-5.68		-8.49			-7.57		-11.14			-6.34		-9.34
t		-5.56		-4.81			-6.14		-9.57			-6.17		-6.25
P		<0.01		<0.01			<0.01		<0.01			<0.01		<0.01

PYLL: potential years of life lost; PYLLR: potential years of life lost rate; SPYLL: standardized potential years of life lost; SPYLLR: standardized potential years of life lost rate.

## 3 讨论

2017至2023年邯郸市居民肺癌死亡率为22.00/10万-28.78/10万，年均死亡率为25.28/10万，标化死亡率为16.58/10万，与济南市的死亡水平相近^[12]^，低于石家庄市的死亡水平^[13]^。根据2020年全国第七次人口普查数据得知^[14]^，邯郸市人口年龄构成与全省相比，偏年轻化，而肺癌属于年龄相关疾病，这是造成邯郸市肺癌死亡率低于全省的主要原因。2017至2023年邯郸市肺癌的标化死亡率呈下降趋势，与南京市研究结果^[15]^一致，说明癌症诊疗技术及居民健康意识提高等多项举措对癌症的防控产生了积极影响。

本研究显示，邯郸市肺癌死亡率随着年龄的增长而增加，85~岁年龄组肺癌死亡率为最高峰，提示年龄是影响癌症发病率和死亡率的关键因素^[16]^，这与既往研究结果相一致^[17,18]^。随着年龄的增加，一方面在环境中接触的致癌物质就越多，另一方面人体机能出现衰退，再者患慢性呼吸系统疾病的概率增加，这些因素相互作用，致使肺癌的死亡风险显著增加^[13,19]^。因此，应重点关注中老年人群，通过开展早期筛查、早期诊断及早期治疗等手段，来降低该年龄段的肺癌死亡率。

本研究发现，2017至2023年邯郸市肺癌CELE呈上升趋势，寿命损失率呈下降趋势，提示因肺癌导致的死亡对邯郸市居民的寿命损失有所减轻，原因可能是控烟政策的大力实施^[20]^、环境污染得到改善、健康生活方式深入民心^[21]^、癌症的早诊早治及肺癌靶向治疗技术的应用，上述因素共同作用提高了全市居民的期望寿命。男性居民的寿命损失率始终高于女性居民，表明男性居民更易暴露于吸烟等危险因素^[22,23]^，烟草烟雾中包含有至少69种致癌物，人体接触这些致癌物后，会使体内关键基因发生永久性突变，且突变逐渐积累，破坏正常生长调控机制，最终引发恶性肿瘤^[24]^。研究^[25,26]^显示，生物学差异、生活方式及行为习惯等因素也可能造成男性寿命损失率高于女性。这些因素的共同作用导致了肺癌死亡及期望寿命存在性别差异。因此，在提升全人群期望寿命的同时，如何进一步缩小男女性别差距，或有望成为未来防治工作的重点方向。

Fulfillment指数是衡量各种死因对各个年龄组人群寿命的影响水平。通过本研究可看出因肺癌导致的Fulfillment指数随年龄增长呈上升趋势，而Fulfillment指数自45~岁、50~岁及55~岁组居民均呈下降趋势，提示可能与该年龄段人群体育锻炼时间相对较长、心肺健康水平较高有关^[27,28]^。

2017至2023年邯郸市因肺癌导致的PYLL为134,219.75人年，SPYLLR为1.49‰，说明肺癌对邯郸市居民的危害程度大，造成了严重的疾病负担。男性PYLLR和SPYLLR高于女性，说明肺癌对男性的寿命损失更大，可能是由于男性吸烟率（50.5%）远超女性（2.1%）^[24]^所致。在我国，肺癌死亡病例中约42.7%（25.1万）由吸烟导致，其中包括22.8万男性和2.3万女性^[29]^，提示吸烟会增加肺癌的死亡风险，因此控制烟草对于防治肺癌尤为重要，且男性是重点关注人群。

本研究存在一定的不足之处：（1）由于数据获取存在局限性，无法全面反映经济发展水平和居民膳食营养状况对期望寿命的影响；（2）死因监测数据由基层及各级医疗卫生机构上报，因基层人员专业能力不足，存在漏报及逻辑性错误，因此，需加强死因监测培训，以进一步提高数据的准确性；（3）本研究数据时间跨度覆盖新型冠状病毒流行期间，但查阅相关文献发现^[30,31]^，相较于其他癌症，新型冠状病毒对肺癌死亡率的影响并无显著差异。影响感染新冠的癌症患者死亡率的主要因素为初级卫生保健系统及癌症治疗情况，且我国癌症一线治疗环境相对欠佳。综合来看，这表明新冠病毒并不会对肺癌死亡率产生直接影响。

综上所述，2017至2023年邯郸市因肺癌导致的疾病负担仍然较为严重。今后需加强男性肺癌防控工作。在控烟及健康生活方式宣传中，应以男性为重点对象，深入工厂、工地等场所，借助广告、自媒体等多种宣传手段，加大控烟宣传力度；强力推进环境整治工作，提高环境质量；积极推动癌症筛查工作，提高肺癌的早诊早治率；同时针对高年龄组人群应采取多种慢性病防治干预措施，从而降低肺癌的死亡率。
